# Runx1 Orchestrates Sphingolipid Metabolism and Glucocorticoid Resistance in Lymphomagenesis

**DOI:** 10.1002/jcb.25802

**Published:** 2017-01-10

**Authors:** A. Kilbey, A. Terry, S. Wotton, G. Borland, Q. Zhang, N. Mackay, A. McDonald, M. Bell, M.J.O. Wakelam, E.R. Cameron, J.C. Neil

**Affiliations:** ^1^ Molecular Oncology LaboratoryInstitute of Infection, Immunity and InflammationUniversity of GlasgowGlasgowG61 1QHUnited Kingdom; ^2^ The Babraham InstituteBabraham Research CampusCambridgeCB22 3ATCambridgeshireUnited Kingdom

**Keywords:** Runx1, ONCOGENE, SPHINGOLIPID, GLUCOCORTICOID, SURVIVAL

## Abstract

The three‐membered *RUNX* gene family includes *RUNX1*, a major mutational target in human leukemias, and displays hallmarks of both tumor suppressors and oncogenes. In mouse models, the *Runx* genes appear to act as conditional oncogenes, as ectopic expression is growth suppressive in normal cells but drives lymphoma development potently when combined with over‐expressed Myc or loss of p53. Clues to underlying mechanisms emerged previously from murine fibroblasts where ectopic expression of any of the *Runx* genes promotes survival through direct and indirect regulation of key enzymes in sphingolipid metabolism associated with a shift in the “sphingolipid rheostat” from ceramide to sphingosine‐1‐phosphate (S1P). Testing of this relationship in lymphoma cells was therefore a high priority. We find that ectopic expression of *Runx1* in lymphoma cells consistently perturbs the sphingolipid rheostat, whereas an essential physiological role for Runx1 is revealed by reduced S1P levels in normal spleen after partial Cre‐mediated excision. Furthermore, we show that ectopic *Runx1* expression confers increased resistance of lymphoma cells to glucocorticoid‐mediated apoptosis, and elucidate the mechanism of cross‐talk between glucocorticoid and sphingolipid metabolism through *Sgpp1*. Dexamethasone potently induces expression of *Sgpp1* in T‐lymphoma cells and drives cell death which is reduced by partial knockdown of *Sgpp1* with shRNA or direct transcriptional repression of *Sgpp1* by ectopic Runx1. Together these data show that *Runx1* plays a role in regulating the sphingolipid rheostat in normal development and that perturbation of this cell fate regulator contributes to Runx‐driven lymphomagenesis. J. Cell. Biochem. 118: 1432–1441, 2017. © 2016 The Authors. *Journal of Cellular Biochemistry* Published by Wiley Periodicals, Inc.

*RUNX1* (*AML1*) is one of the most frequently affected genes in human leukemias where it is subject to mutational processes including chromosomal translocation, gene amplification, and point mutation [Osato, 2004; De Braekeleer et al., 2009]. In acute myeloid leukemias, diverse chromosomal breakage and fusion events result in truncation of the *RUNX1* gene product and deletion or replacement of the C‐terminal transactivation domain with heterologous sequences. Point mutation with apparent loss of function or dominant negative activity is a feature of immature M0 AML [Schnittger et al., 2011] suggesting that RUNX1 may act as a tumor suppressor in this lineage and that the oncogenic fusions operate primarily by a dominant negative mechanism [De Braekeleer et al., 2009]. A tumor suppressor role for *Runx1* in myeloid leukemia is also suggested by mouse models where Runx1 deletion is induced, for example, in Flt3‐ITD expressing mice [Mead et al., 2013]. However, the notion that *RUNX1* is simply a suppressor whose loss of functions confers an automatic growth advantage is challenged by more recent observations that human leukemia cells bearing the common RUNX1‐ETO fusion cannot tolerate loss of the remaining wild‐type *RUNX1* allele [Ben‐Ami et al., 2013]. Moreover, although the frequent *TEL‐RUNX1* fusion in childhood B‐ALL is often complemented by loss of the wild‐type *TEL* allele, the unaffected *RUNX1* allele is generally intact and is in fact more likely to show copy number gain [Niini et al., 2000].

Early evidence from mouse models showed that all three members of the Runx family can act as targets for transcriptional activation in retrovirus‐induced lymphomas [Stewart et al., 2002, 1997; Wotton et al., 2002]. Moreover, transgenic over‐expression leads to predisposition to lymphoma and is strongly synergistic with other oncogenes (*Myc*, *Myb*, *Pim1*) or loss of p53 tumor suppressor function [Vaillant et al., 1999; Blyth et al., 2001, 2006; Shimizu et al., 2013; Huser et al., 2014]. Although oncogenic activity has been most extensively documented for Runx2, ectopic expression of Runx1 has been shown to collaborate strongly with Myc in T and B‐cell lymphomagenesis [Blyth et al., 2009]. Although a small proportion of primary human T‐cell acute lymphoblastic leukemias (ALL) carry point mutations in RUNX1 [Grossmann et al., 2011], evidence for an oncogenic role for this gene in childhood B‐ALL is suggested by the high expression of *RUNX1* [Niini et al., 2000] and high level amplification in a poor prognosis subset [Robinson et al., 2003]. These findings reinforce the hypothesis that the Runx gene family can operate as tumor suppressors or as oncogenes depending on the context in which misregulation occurs.

Clues to the contextual factors that influence the outcome of Runx gain or loss have come from studies in mouse and human fibroblasts where integrity of the p53 pathway determines the response to ectopic Runx expression. Normal primary fibroblasts undergo senescence‐like growth arrest in response to ectopic Runx expression, while cells in which the p53 pathway is disabled instead display enhanced survival and oncogenicity [Wotton et al., 2004; Kilbey et al., 2007; Wolyniec et al., 2009]. Moreover, ectopic Runx expression in immortalized *Ink4a/Arf* null fibroblasts revealed a novel link between oncogenic transcription factors and sphingolipid metabolism [Wotton et al., 2008]. Several enzymes involved in sphingolipid metabolism (*Sgpp1*, *Ugcg*, *Siat9*) were identified as direct Runx targets and regulated in a manner consistent with elevated Sphingosine‐1‐phosphate (S1P) and reduced ceramide levels, changes which were also confirmed at the biochemical level [Wotton et al., 2008; Kilbey et al., 2010].

Bioactive lipids mediate many essential roles in normal development and disease. S1P is among the best characterized and is recognized as a regulator of key processes in cancer development including survival, proliferation, and angiogenesis [Takabe et al., 2008]. The precursors of S1P, sphingosine, and ceramide are mediators of cell growth arrest and apoptosis [Zhang et al., 1991; Olivera and Spiegel, 1993; Cuvillier et al., 1996]. Regulation of the balance between these interconvertible lipids forms the basis of the sphingolipid rheostat and the means by which ceramide, sphingosine, and S1P control cell growth and survival in response to environmental and cellular cues. Multiple enzymes control the rheostat and the biochemical processes that generate or degrade its key components [Van Brocklyn and Williams, 2012]. However, despite the wealth of literature on the biology and biochemistry of these processes, links between sphingolipid signaling and cancer gene function, have emerged only recently [Kilbey et al., 2010; Heffernan‐Stroud et al., 2012; Burns et al., 2013; Kajiwara et al., 2014]. The evidence of Runx regulation of sphingolipid metabolism in fibroblast survival stimulated our interest in its relevance to lymphomagenesis where there is strong evidence for an oncogenic role. We examined cell lines from p53 null and Eμ‐Myc lymphomas where ectopic Runx expression has been shown to collaborate strongly in oncogenesis [Blyth et al., 2009, 2001]. Our results demonstrate a consistent role for Runx1 in control of sphingolipid metabolism in lymphomas as well as in normal development. Moreover, we show that Runx1 regulates glucocorticoid sensitivity in lymphoma cells and identify an established direct target gene, *Sgpp1*, as an integrator of sphingolipid metabolism and responses to glucocorticoids.

## MATERIALS AND METHODS

### GENERATION OF TRANSGENIC MICE AND CELL LINES

MxLoxP mice (Mx1Cre^+^ *Runx1^fl/fl^*) were created as previously described [Borland et al., 2016]. Mx1Cre positive and negative offspring were treated at seven weeks of age with six injections of 600 μg polyinosinic polycytidylic acid (pIpC), or an equivalent volume of PBS, on alternate days and sacrificed for tissue samples two days after the final pIpC treatment.

Tumor‐prone Runx1‐floxed mice carrying the *EμMyc* transgene and heterozygous for *p53* loss (Mx1Cre^*+*^Runx1^fl/fl^EμMyc^+^p53^+/−^) were created by crossing Mx1Cre^*+*^Runx1^fl/fl^p53^+/−^ mice with *Runx1^fl/fl^EμMyc* ^+^ mice as previously described [Borland et al., 2016]. Offspring were sacrificed at onset of tumor symptoms and cells from splenic tumors placed into culture to establish cell lines.

Animal protocols used in this work were evaluated and approved by the University of Glasgow Ethics and Welfare Committee and were carried out under Home Office License (approval granted September 2012, license number PPL 60/4408) as governed by the Animal Scientific Procedures Act, 1986 and EU Directive 2010.

### CELL CULTURE AND TRANSFECTIONS

Cell lines were established from p53^−/−^ thymic lymphomas or EμMyc splenic lymphomas as previously described [Borland et al., 2016]. Transfections were carried out using Superfect Transfection Reagent (Qiagen, Crawley, UK) according to the manufacturer's instructions. Conditions for transfection have been previously described (21). The 3s cell line was derived from an Mx1Cre^+^ *Runx1^fl/fl^EμMyc* ^+^ *p53* ^+/−^ tumor treated with and without 10U/ml IFNβ (R&D systems) for 8 h to excise *Runx1*. For growth analyses cells were plated at 5 × 10^5^/ml (T cell lymphoma) or 2.5 × 10^5^/ml (B cell lymphoma) in a 12‐well plate in triplicate. Live/dead cell counts were carried out using a hemocytometer and Trypan blue as a vital indicator.

### PCR GENOTYPING PROTOCOLS AND PRIMERS

Tissue DNA samples were prepared using the Nucleon Genomic DNA Extraction Kit. Runx1 primers for WT, floxed and deleted alleles, and PCR cycling conditions are as described in Chen et al. [2009]. The PCR products were resolved on a 2.0% TAE agarose gel. Control DNAs includes Balb/c kidney (WT Runx1) and Mx1Cre *Runx1^fl/fl^* spleen (non‐excised floxed Runx1, excised Runx1).

### QUANTITATIVE REAL‐TIME PCR

Runx1‐expressing and control lymphocytes were plated in triplicate on 6 well plates at 5 × 10^6^/well in the presence and absence of 1.0 μM dexamethasone for 6 h. RNA extraction and cDNA preparation were performed as described (21). For quantitative real‐time PCR, 12.5 ng aliquots of cDNA were amplified in triplicate using primers for murine endogenous control *Hprt* or primers for murine *Sgpp1*, *Siat9*, *Nr3c1*, *Runx1* (Qiagen QuantiTect Primer Assays) or *Ugcg* (779F 5′ tttgctcagtacattgctgaagatta 3′ and 861R 5′ acttgagtagacattgaaaacctccaa 3′). Relative quantification was carried out and calibrated to vector control samples (18).

### LENTIVIRUS PRODUCTION AND GENE KNOCKDOWN

SMARTvector 2.0 lentiviral shSgpp1 and shNC non‐coding control particles were purchased from Thermo Scientific (2 × 10^8^ particles/ml). shGAPDH lentiviral particles were included as a positive control for transduction efficiency. Virus supernatants were thawed on the day of transfection and centrifuged onto retronectin‐coated plates at 3220 g for 1 h at 4°C (4 × 10^5^ particles/well of a 24‐well plate). Supernatants were aspirated from the plates and replaced with fresh supernatant (2 × 10^5^ particles/well) and spun as before. The 10^5^ cells were added in an equal volume per well and incubated for 7 h with 8 μg/ml polybrene. The medium was replaced with fresh medium overnight and then removed. A second infection with 2 × 10^5^ virus particles in an equal volume of fresh medium containing 8 μg/ml polybrene was performed for a further 7 h before the cells were dissociated from the retronectin and incubated in fresh medium. After 40 h, the medium was replaced with media containing 2 μg/ml puromycin to allow for selection of virally transduced cells. For qt‐RT‐PCR and viability counts, puromycin‐resistant cells were plated as described in the presence and absence of 1.0μM dexamethasone.

### MASS SPECTROMETRY: CERAMIDE AND S1P EXTRACTION AND ANALYSIS FROM CELLS

Ceramide and S1P extraction was performed with a modified Folch approach. Briefly, cells from pellets or pulverized tissues were washed twice with cold PBS and re‐suspended in 1.5 ml methanol. After addition of 50 ng C17‐ceramide and 15 ng C17‐S1P as internal standards, 1.5 ml of 0.88% NaCl and 3 ml chloroform were added, the mixture vortexed for 20 s and sonicated for 2 min. Then the sample was centrifuged at 1000*g* at 4C for 15 mins. The lower phase was collected. The upper phase was re‐extracted with 3 ml synthetic lower phase (mix chloroform/methanol/0.88% NaCl at volume ratio of 2:1:1, after phase separation, take the lower phase as synthetic lower phase). The lower phases were combined, dried with SpeedVac (Thermo) and re‐dissolved in 60 μl chloroform. A total of 7 μl were injected for LCMS/MS analysis. Another 7 μl of sample was used for S1P analysis as described below with 4000QTRAP (AB Sciex). For LC/MS/MS analysis of ceramide, a Thermo Orbitrap Elite system (Thermo Fisher) was hyphenated with a five‐channel online degasser, four‐pump, column oven, and autosampler with cooler Shimadzu Prominence HPLC system. Lipid classes were separated on a normal phase silica gel column (2.1 × 150 mm, 4 micro, MicoSolv Technology) with hexane/dichloromethane/chloroform/methanol/acetanitrile/water/ethylamine solvent gradient based on the polarity of head group. High resolution (240 k at m/z 400)/accurate mass (with mass accuracy <5 ppm) and tandem MS (CID fragmentation) were used for molecular species identification and quantification and further confirmed by reference to appropriate lipids standards. Orbitrap Elite mass spectrometer operation conditions: for ceramide analysis, heated ESI Source in negative ESI mode, heater temperature: 325°C, sheath gas flow rate (arb): 45, aux gas flow rate (arb): 10, sweep gas flow rate (arb): 0, I spray voltage: 3.0 kV, capillary temperature: 375°C, S‐Lens RF level: 70%. Orbitrap mass analyzer was operated as SIM scan mode with mass range: m/z 510–740, mass resolution: 240 k at m/z 400. Ion trap mass analyzer was operated as dependent scan in CID mode at normal scan speed.

### MASS SPECTROMETRY: S1P EXTRACTION AND ANALYSIS FROM CELL CULTURE MEDIA

A total of 800 μl cell culture media, 200 μl 0.88% NaCl, and 2 ml n‐butanol were mixed in pre‐cleaned 1.5 ml Eppendorf tubes, with 15 ng C17‐S1P as internal standard. After vortexing for 20 s at room temperature and sonication in an ice‐cold water bath for 3 min, the tubes were centrifuged at 1500*g* 4°C for 10 min collecting the upper organic phase. The lower phase was re‐extracted with 2 ml n‐butanol and the organic extractions combined. The lipid extract was dried under vacuum at room temperature with SpeedVac (Thermo), re‐dissolved in 60 μl chloroform/methanol/water 2:5:1 (v/v/v) solvent mixture. A total of 7 μl were injected onto a Gemini—NX—3u C18 2.0 × 150 mm column (Phenomenex) for LC‐MS/MS analysis of S1P with Shimadzu Prominence HPLC system hyphenated with AB Sciex 4000 QTRAP mass spectrometer. The S1P separation was carried out with a binary gradient elution at flow rate of 0.25 ml/min with column oven set at 25°C. Mobile phase A: 5% acetonitrile with 1% formic acid and 5 mM ammonium formate; Mobile phase B: 95% acetonitrile with 1% formic acid and 5 mM ammonium formate. The gradient was run from 10% B to 100% B in 10 min. A total of 4000 QTRAP mass spectrometer source/gas parameters: curtain gas: 25; collision gas: high; ionspray voltage: −4500; temperature: 550; ion source gas 1: 30; ion source gas 2: 45; interface heater: on. The following Compound Parameters were used for Multiple Reaction Monitoring (MRM) analysis of each molecular species of S1P. Both Q1 and Q3 mass were set up at unit resolution (Table I).

**Table I:** Compound Parameters for Multiple Reaction Monitoring

Compound	Q1 mass (Da)	Q3 mass (Da)	Declustering potential(DP)	Entrance potential (EP)	Collision energy (CE)	Collision cell exit potential (CXP)
C16‐S1P	350.2	78.9	−90	−10	−48	−5
C17‐S1P	364.2	78.9	−90	−10	−48	−5
C18:1‐S1P	376.2	78.9	−95	−8	−62	−5
C18‐S1P	378.2	78.9	−95	−8	−62	−5
C20‐S1P	406.3	78.9	−95	−8	−62	−5

### WESTERN BLOTTING AND ANTIBODIES

Preparation of whole cell protein extracts was performed as described previously (21). Samples equivalent to 30 μg of protein (Bio‐Rad protein assay) were resolved on 8% SDS polyacrylamide gels and transferred to enhanced chemiluminescence nitrocellulose membranes (ECL; Amersham, Little Chalfont, UK). The antibodies used were α Runx1 #8229 (Cell Signalling Technologies) and α actin sc1616 (Santa Cruz).

### STATISTICAL ANALYSIS

Graphs were generated with Excel as mean values and error bars relate to standard deviations. All statistical comparisons were performed using the Student's *t* test. Unless stated a significance value = <0.05 is denoted by (*) and of = <0.01 by (**).

## RESULTS

### Runx1 PROMOTES S1P RELEASE FROM LYMPHOMA CELLS

**Figure 1:**
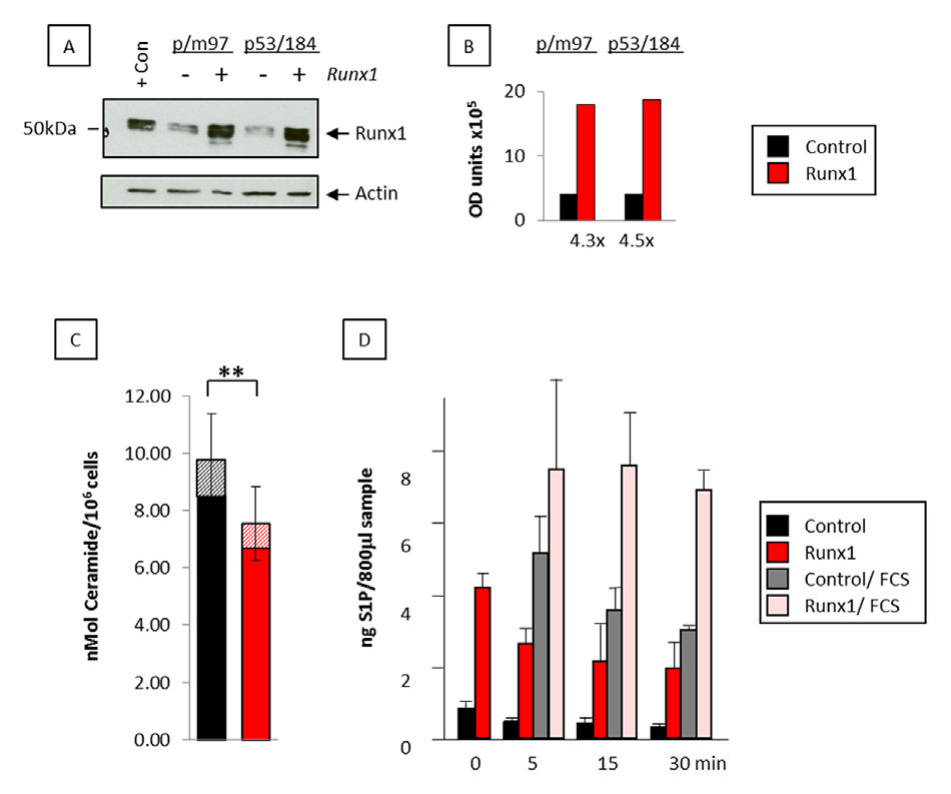
Ectopic Runx1 promotes S1P release from T‐lymphoma cells. (A) Total protein was extracted from p/m97 or p53/184 thymic lymphoma cells transduced with the pBabeRunx1 retroviral vector or the pBabePuro vector control and probed against antibodies to Runx1 (Cell Signalling #8229) or actin (Santa Cruz sc‐1616) as a loading control. Lymphoma cells over‐expressing Runx1 (9) were included as a positive control. (B) The blot was quantified using image J software and the Runx1 fold change indicated below the histogram. (C) Long chain ceramides were extracted from cell pellets from pBabePuro vector control and pBabeRunx1‐expressing T lymphoma cells (p/m97 shown), and separated, identified, and semi‐quantitated by HPLC mass spectrometry. The data are means ± SD where n = 4 from one experiment typical of two. Solid bars represent combined levels of 16.0, 24.1, and 24.0‐Cer (*P* = <0.01). A significant difference between ectopic Runx1 expressing and non‐expressing cells was preserved when the remaining ceramides (14.0, 16.1, 18.0, 20.0, 22.1, 24.2‐Cer—displayed in hashed bars) were included in the total (*P* = <0.01). (D) C18‐S1P was analyzed from conditioned media collected from pBabePuro vector control and pBabeRunx1‐expressing T lymphoma cells by HPLC mass spectrometry (p/m97 shown). FCS was included to activate Sphk1. The data are means ± SD where n = 4 from one experiment typical of two.

We previously reported that ectopic Runx1 depletes intracellular long chain ceramides and opposes ceramide‐induced apoptosis in murine fibroblasts [Kilbey et al., 2010]. To determine whether the sphingolipid rheostat is similarly perturbed in a context where Runx1 has established in vivo oncogenic function [Blyth et al., 2001; Shimizu et al., 2013], we introduced Runx1 into two independently derived p53‐null T cell lymphoma lines (Fig. 1A). Vector‐driven expression led to increased levels of Runx1 protein at 4.3‐ and 4.5‐fold over the endogenous levels in p/m97 and p53/184 cells, respectively (Fig. 1B). Mass spectrometric analysis of ceramide/S1P revealed modest but consistent reductions in the levels of the most abundantly expressed long chain intracellular ceramides (16.0, 24.1, and 24.0‐Cer) in the presence of ectopic Runx1 (Fig. 1C—solid bars *P* = <0.01). The remaining ceramides (14.0, 16.1, 18.0, 20.0, 22.1, 24.2‐Cer; Fig. 1C—hashed bars) were detected at much lower levels but a significant reduction was preserved when these were included in the total (*P* = <0.01). A more dramatic effect was observed on extracellular C18‐S1P which was induced three to fivefold in the presence of ectopic Runx1 (Fig. 1D). The level of induction was further stimulated by serum (FCS) to activate sphingosine kinase and the conversion of sphingosine to S1P. Indeed by 15 min, serum‐induced levels of C18‐S1P in the presence of ectopic Runx1 (pink bar) exceeded the sum of ectopic Runx1 (red bar) or FCS (gray bar) alone suggesting that ectopic Runx1 and FCS have additive effects in T lymphoma cells and that absolute levels of Runx1 modulate C18‐S1P production in vitro.

### ENFORCED DELETION OF Runx1 IMPAIRS S1P RELEASE IN VIVO

**Figure 2:**
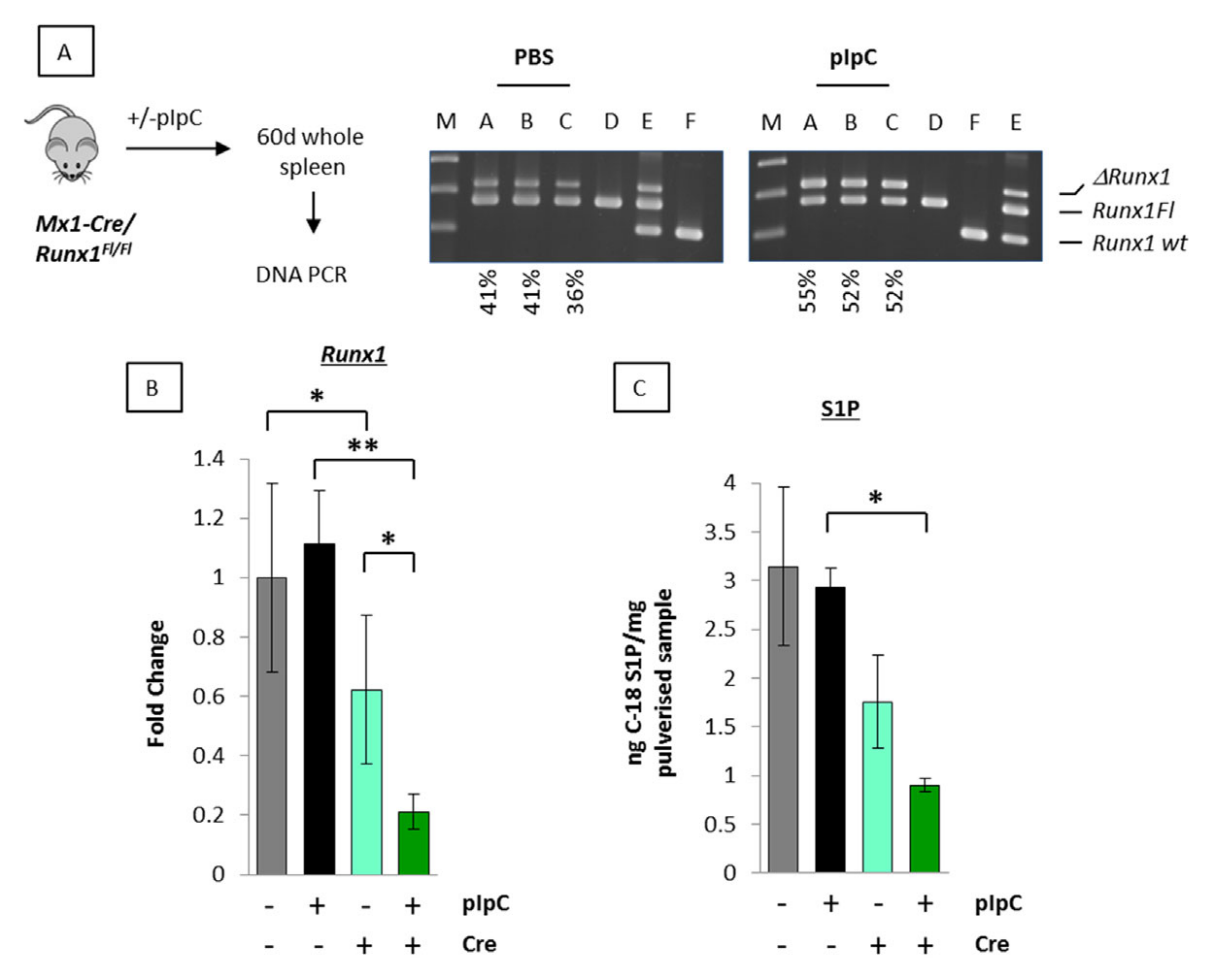
Enforced deletion of *Runx1* impairs S1P release in vivo. (A) PCR genotyping of 60‐day‐old splenic tissue DNA from *Runx1^fl/fl^*Mx1Cre^+^ mice treated with vehicle control (PBS) for background excision or pIpC to excise *Runx1*. Samples: A–C, 60‐day spleen tissue samples from two groups of three *Runx1^fl/fl^*Mx1Cre^+^ mice treated with either PBS or pIpC; D–F DNA controls, non‐excised *Runx1^fl/fl^*Mx1Cre^−^ (D), a mixture of partially excised *Runx1^fl/fl^*Mx1Cre^+^ and Balb/c normal kidney to show all three possible PCR products (E) and Balb/c normal kidney (F). Positions of Floxed (Runx1Fl), deleted (ΔRunx1Fl), and wildtype (WT) *Runx1* alleles are as shown. Quantitation to estimate relative excision was performed using image J software. Estimated background and pIpC excision rates are labeled under the blots. (B) qt‐RT‐PCR analysis of *Runx1* expression in 60‐day splenic tissue RNA samples from *Runx1^fl/fl^*Mx1Cre^+^ mice treated with vehicle control or pIpC (dark and light green bars, respectively). Parallel samples were analyzed from *Runx1^fl/fl^* mice lacking the Mx1Cre gene to control for PBS and pIpC treatment (black and gray bars, respectively). The data are means ± SD where n = 9 representing three technical replicates of each biological replicate (3). A significant reduction in *Runx1* expression was observed between Mx1Cre^+^ and Mx1Cre^−^ mice indicating background excision (gray vs. light green bar; *P* = <0.05). Inclusion of pIpC to excise *Runx1* generated a further reduction (black vs. green bar; *P* = <0.01). (C) Remaining splenic tissues were pulverized and analyzed by HPLC mass spectrometry for C18‐S1P. Data are expressed relative to 1 mg of pulverized spleen tissue and represent means ± SD of spleens of three mice for each set of conditions.

To determine whether Runx1 plays an essential physiological role in sphingolipid metabolism in vivo we attempted to delete *Runx1* in otherwise genetically normal *Runx1^fl/fl^Mx1Cre* ^+^ conditional knockout mice. A previously validated direct PCR assay was used to measure the proportion of excised to non‐excised allele in the tissue [Borland et al., 2016]. As reported, most normal mouse tissues resist enforced deletion of *Runx1* [Tober et al., 2013], suggesting that this gene plays an essential maintenance role in many mature cell types. Nevertheless, we were able to achieve partial *Runx1* ablation in spleen tissues from 60‐day‐old *Runx1^fl/fl^*Mx1Cre^+^ transgenic animals where pIpC induction of Cre recombinase led to *Runx1* excision rates of ∼50–55%. Background excision in Mx1Cre^+^ mice treated with PBS alone was in the order of 40% presumably due to endogenous interferon activity (Fig. 2A). *Runx1* transcript levels as measured by qt‐RT‐PCR closely paralleled DNA excision rates in Mx1Cre^*+*^ spleens (pale green vs. dark green bars) and were significantly different from control *Runx1^fl/fl^* mice lacking Mx1Cre that showed no response to pIpC treatment (Fig. 2B; gray vs. black bars). Despite these substantial excision rates, Runx1‐excised mice showed surprisingly little perturbation in spleen cell populations [Borland et al., 2016]. Parallel tissue samples were processed for biochemical analysis of C18‐S1P by mass spectrometry and revealed a strong correlation between loss of Runx1 and C18‐S1P in vivo (Fig. 2C). Splenic tissue levels of C18‐S1P were moderately reduced by background excision alone (gray bars vs. pale green bars), but significant depletion was observed in the presence of pIpC (black bars vs. dark green bars). To control for non‐specific effects of the Cre recombinase spleen tissue samples were prepared from *Runx1^wt/wt^*—Mx1Cre^+^ and *Runx1^wt/wt^*—Mx1Cre^−^ transgenic mice treated with and without pIpC as described previously. In this case, no significant differences in C18‐S1P were recorded between the different experimental groups (data not shown), suggesting that loss of *Runx1* was responsible for the reduced levels of C18‐S1P in pIpC‐treated *Runx1^fl/fl^*Mx1Cre^+^ mice.

### Runx1 PROTECTS LYMPHOMA CELLS AGAINST DEXAMETHASONE‐INDUCED APOPTOSIS

**Figure 3:**
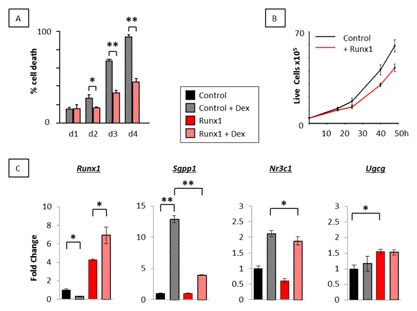
Runx1 protects lymphoma cells against dexamethasone‐mediated apoptosis. (A) p53 null lymphoma cells transduced with the pBabeRunx1 retroviral vector or the pBabePuro vector control (p/m97 shown) were plated in triplicate in the presence and absence of 1.0 μM dexamethasone and monitored for live/dead counts by trypan blue exclusion over 4 days. (B) The same cells were plated at 4 × 10^5^ per well in triplicate wells of a 12‐well plate and monitored for growth over 48 h. Ectopic Runx1 significantly reduced cell proliferation at 24 h (*P* < 0.01), 40 h (*P* < 0.01), and 48 h (*P* < 0.01). (C) qt‐RT‐PCR analysis of *Runx1*, *Sgpp1*, *Ugcg*, and *Nr3c1* in pBabePuro vector control and pBabeRunx1‐expressing T lymphoma cells (p/m97 shown) grown in the presence and absence of 1.0 μM dexamethasone for 6 h. The data are means ± SD where n = 9 representing three technical replicates of each biological replicate (3) from one experiment typical of two.

We previously reported that each of the three *Runx* genes can oppose dexamethasone‐induced growth inhibition in murine fibroblasts, at least in part by down‐regulation of the glucocorticoid receptor gene, *Nr3c1* [Wotton et al., 2008]. We tested this observation further in lymphoid cells where dexamethasone induces apoptosis rather than growth stasis and glucocorticoids feature as important therapeutic agents in lymphoma treatment [Bhadri et al., 2012]. Using trypan blue exclusion as a surrogate for viability, ectopic Runx1 was observed to provide a sustained protective effect against dexamethasone that increased markedly from 48 h after dexamethasone exposure (Fig. 3A). The protective effect was even more striking in light of the modest growth inhibition observed with ectopic Runx1 in this cell background (Fig. 3B). Together these data suggest that protection against dexamethasone‐induced death is due to increased survival mediated by Runx1. Modulation of pro‐ and anti‐apoptotic enzyme expression to promote apoptosis was previously reported in response to dexamethasone in double positive murine thymocytes [Bianchini et al., 2006]. Of these, *Sgpp1* and *Ugcg* were also identified as direct transcriptional targets of Runx1 in murine fibroblasts, whereas the glucocorticoid receptor *Nr3c1* was identified as an indirect target [Wotton et al., 2008; Kilbey et al., 2010]. To determine whether Runx1 opposed dexamethasone‐mediated gene expression to promote cell survival we examined transcription of *Runx1*, *Sgpp1*, *Ugcg*, and *Nr3c1* by qt‐RT‐PCR in the presence and absence of dexamethasone and ectopic Runx1. As shown in Figure 3C, endogenous *Runx1* was repressed by dexamethasone as previously reported, but the pattern was reversed in the presence of ectopic Runx1. In contrast, *Sgpp1* expression was dramatically opposed by ectopic Runx1 in dexamethasone‐treated p/m97 cells whereas *Nr3c1* expression was not. Indeed, as previously reported in murine fibroblasts, *Nr3c1* was slightly downregulated by ectopic Runx1 but was upregulated by dexamethasone in both the presence and absence of ectopic Runx1 [Wotton et al., 2008]. *Ugcg* expression was refractory to dexamethasone treatment in p53‐null thymic lymphoma cells but was induced by Runx1. This finding contrasts with double positive murine thymocytes where *Ugcg* was reportedly repressed by dexamethasone [Bianchini et al., 2006] but was consistent with the effects of ectopic Runx1 on *Ugcg* transcription in murine fibroblasts [Wotton et al., 2008; Kilbey et al., 2010]. Together the data support a mechanism whereby the opposition of *Sgpp1* transcription and the overexpression of *Ugcg* might contribute to Runx1‐induced survival in the presence of dexamethasone in thymic lymphoma cell lines.

### Runx1 EXCISION PROMOTES DEXAMETHASONE‐INDUCED APOPTOSIS AND Sgpp1 TRANSCRIPTION

**Figure 4:**
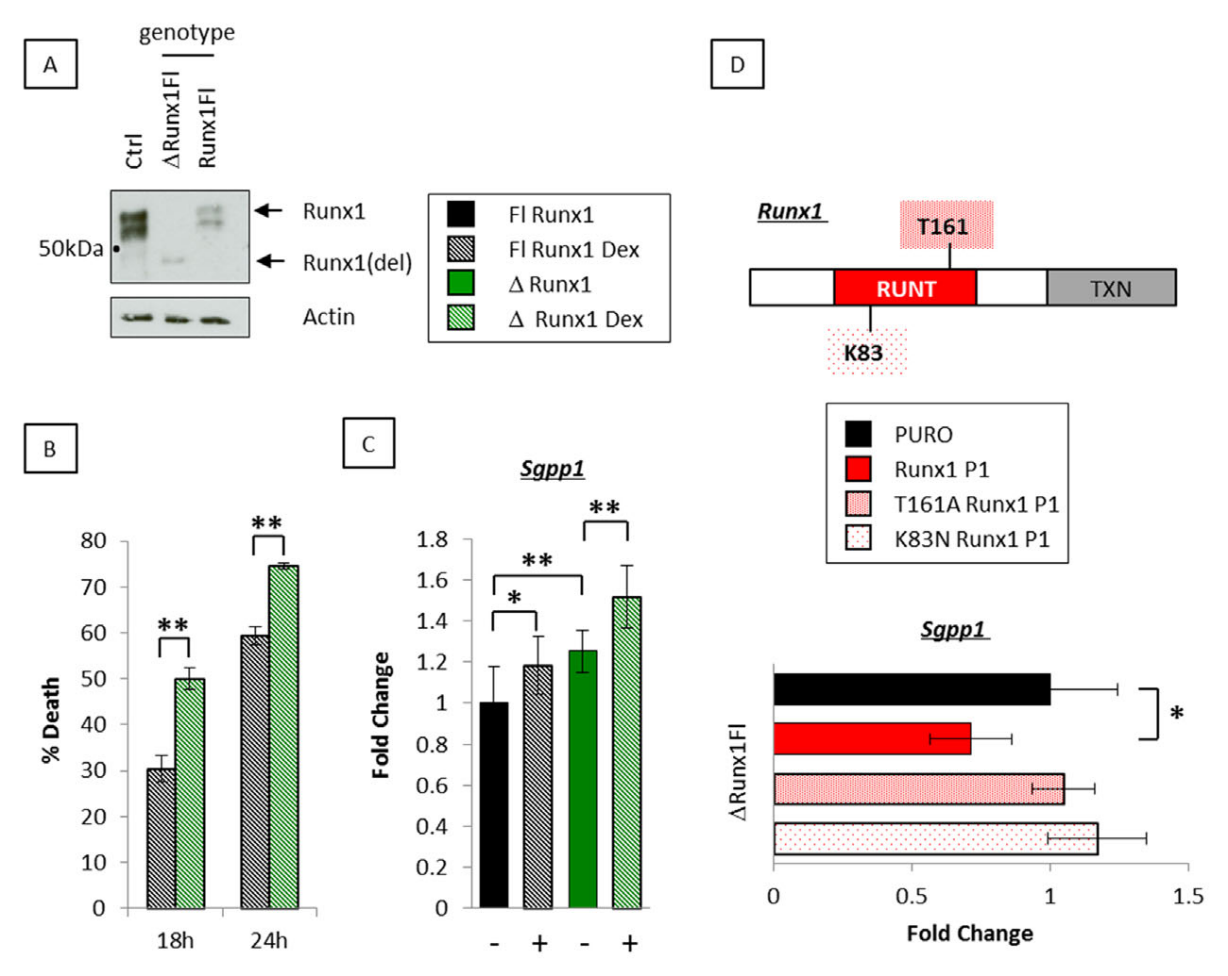
Enforced deletion of *Runx1* promotes dexamethasone‐mediated apoptosis and *Sgpp1* transcription. (A) Western blotting analysis as described in Figure 1A to detect the deleted (ΔRunx1Fl) and full length (Runx1Fl) Runx1 proteins from in vitro excised in *Runx1^fl/fl^*Mx1Cre^+^ 3s B lymphoma cells. (B) Paired cell lines expressing the deleted (ΔRunx1Fl) and full length (Runx1Fl) proteins after in vitro excision of *Runx1^fl/fl^*Mx1Cre^+^ 3s B lymphoma cells were plated in triplicate in the presence of 1.0 μM dexamethasone and monitored for live/dead counts by trypan blue exclusion. (C) qt‐RT‐PCR analysis of steady state levels of *Sgpp1* in ΔRunx1Fl and Runx1Fl 3s cells grown in the presence and absence of 1.0 μM dexamethasone for 6 h. The data are means ± SD where n = 9 representing three technical replicates of each biological replicate (3) from one experiment typical of two. (D) Runx1 schematic showing the mutated residues in the heterodimerization (T161A) and DNA‐binding (K83N) domains. qt‐RT‐PCR analysis of *Sgpp1* expression in ΔRunx1Fl 3s cells transfected with full length Runx1, T161A Runx1, or K83N Runx1. Absolute levels of *Sgpp1* were compared to control cultures expressing the pBabe Puro vector alone. The data were compiled as described in (C).

To further explore the relationship between Runx1 and Sgpp1 in dexamethasone‐induced apoptosis, we examined *Runx1^fl/fl^/*Mx1Cre^*+*^ B cell lymphoma lines established from a mouse model heterozygous for p53 loss and expressing c‐Myc in the B cell compartment (Eμ‐Myc). We exploited interferon β induction of Mx1Cre to mediate in vitro excision of *Runx1* and derived a pair of clonally identical daughter cell lines that express low but readily detectable levels of full length Runx1 (Runx1Fl) or the deleted non‐functional variant (ΔRunx1Fl—Fig. 4A) and asked whether loss of Runx1 promotes dexamethasone‐induced apoptosis and *Sgpp1* transcription. Enforced deletion of endogenous *Runx1* significantly increased cell death in response to 1.0 μM dexamethasone (Fig. 4B). Moreover, enforced deletion of *Runx1* increased expression of *Sgpp1* in ΔRunx1Fl cells (black vs. green bars) and this was further increased in the presence of dexamethasone (hashed black vs. green bars) (Fig. 4C). Parallel cell lines derived from *Runx1^wt/wt^/*Mx1Cre^+^/*p53^+/−^*/Eμ‐Myc lymphomas were refractory to interferon β with respect to *Sgpp1* and *Runx1* expression [Borland et al., 2016] thereby excluding a non‐specific effect of the Mx1Cre recombinase as a causative factor for the increase in *Sgpp1* expression following *Runx1* excision. To determine which functional domains of Runx1 are required to suppress *Sgpp1* induction in the presence of dexamethasone, we introduced a panel of Runx1 mutants (Fig. 4D) into ΔRunx1Fl cells which essentially lack Runx1 expression. qt‐RT‐PCR analysis revealed that Runx1 repression of *Sgpp1* was dependent on intact DNA binding and heterodimerization domains (Fig. 4D), indicating that Runx1 directly opposes dexamethasone‐induced transcription of *Sgpp1*.

### Sgpp1 KNOCKDOWN REDUCES DEXAMETHASONE‐INDUCED CELL DEATH

**Figure 5:**
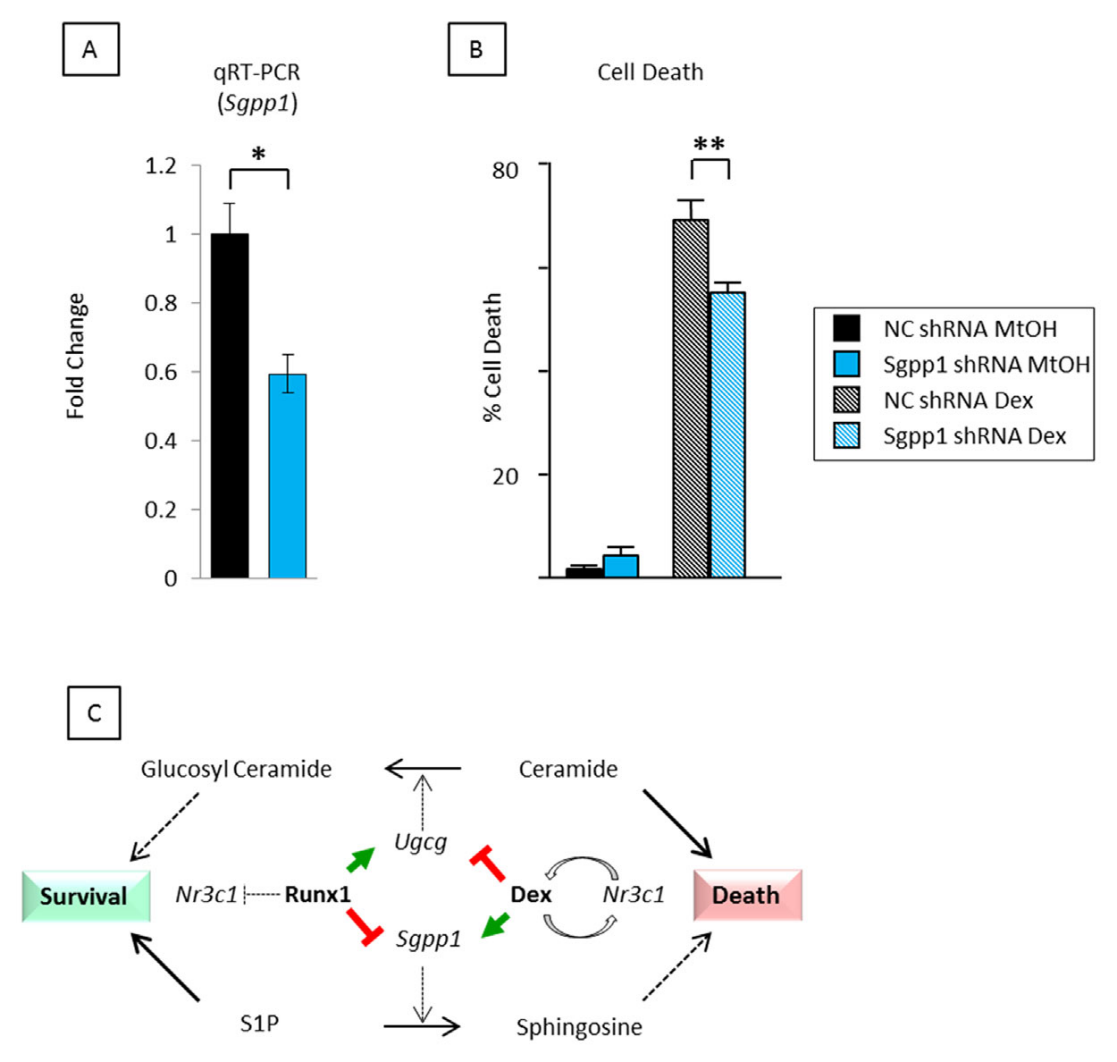
shRNA knockdown of *Sgpp1* reduces dexamethasone‐mediated apoptosis. (A) qt‐RT‐PCR analysis of *Sgpp1* expression in p/m97 cells stably infected with viral supernatants expressing an *Sgpp1* or a non‐coding (NC) control shRNA sequence. Cells were grown for 6 h in the presence of 1.0 μM dexamethasone prior to RNA extraction and qt‐RT‐PCR analysis. The data were calculated as described in Fig. 4C. (B) The same cells were plated in triplicate and grown for 36 h in the presence (Dex) and absence (MtOH) of 1.0 μM dexamethasone and monitored for live/dead counts by trypan blue exclusion. Knockdown of *Sgpp1* had no effect on cell viability under control conditions but gave a significant reduction in cell death in the presence of 1.0 μM dexamethasone. (C) Interplay between Runx1 and dexamethasone on the expression of sphingolipid metabolism enzymes involved in the synthesis and breakdown of sphingosine and ceramide and their potential contributions to cell death and survival [Bianchini et al., 2006; Kilbey et al., 2010].

To investigate the role of *Sgpp1* as a direct mediator of glucocorticoid‐mediated apoptosis we used a short hairpin (sh) RNA to knock down expression of *Sgpp1* in p/m97 thymic lymphoma cells which displayed the most dramatic induction of *Sgpp1* in response to dexamethasone (Fig. 3C—black bar vs. hashed black bar). Retroviral transduction of *Sgpp1* shRNA viral supernatants reduced dexamethasone‐induced expression of *Sgpp1* by ∼40% compared to the non‐coding (NC) shRNA control sequences (Fig. 5A). Stably transduced cells were then cultured in the presence and absence of dexamethasone and cell death measured by trypan blue exclusion. After 36 hr, dexamethasone‐induced death was reduced by ∼20% in cells receiving *Sgpp1* shRNA compared to the NC shRNA control sequence (Fig. 5B). Together these data indicate that Sgpp1 induction is at least partially responsible for death of lymphoma cells mediated by glucocorticoid signaling. Insights gained from this data regarding the interplay between dexamethasone and Runx1 for the regulation of sphingolipid metabolism and cell survival are illustrated in the model shown in Figure 5C.

## DISCUSSION

Previous studies in murine fibroblasts identified three sphingolipid metabolism enzymes as direct transcriptional targets of the three members of the Runx gene family [Wotton et al., 2008; Kilbey et al., 2010]. Moreover, the pronounced pro‐survival effects of ectopic Runx expression in this context were associated with depletion of intracellular ceramide levels suggesting a shift in the sphingolipid rheostat in favor of pro‐survival S1P [Kilbey et al., 2010]. We have now extended these studies to the lymphoid system where ectopic Runx expression has been shown to be potently oncogenic in combination with other cancer gene lesions including p53 loss and Myc over‐expression [Vaillant et al., 1999; Blyth et al., 2001, 2006; Shimizu et al., 2013]. In thymic lymphoma cells Runx1 expression consistently regulates sphingolipid metabolism to favor S1P production with concomitant reduction in long chain ceramides. As we found, no positive effect of Runx1 over‐expression on cell proliferation in this cell background, these results strongly reinforce previous evidence for enhanced survival as a key mechanism in Runx oncogenesis [Blyth et al., 2006]. Moreover, we have shown that partial deletion of Runx1 in vivo results in a significant and specific reduction in tissue S1P levels, revealing a fundamental physiological role for Runx regulation of sphingolipid metabolism in normal hematopoiesis. The production and distribution of S1P in spleen is known to be tightly regulated [Ramos‐Perez et al., 2015] and the importance of Runx1 expression for maintenance of S1P levels described here is likely to be critical for regulation of the immune system.

The regulation of sphingolipid metabolism by the Runx genes is a multigenic phenomenon involving both direct and indirect gene regulation and entails a series of incremental changes in individual genes that combine to achieve significant metabolic shifts [Wotton et al., 2008; Kilbey et al., 2010]. However, this study highlights an additional need to measure transcriptomic changes under stress if the consequences of Runx modulation for sphingolipid metabolism are to be fully understood. In particular, ectopic Runx1 significantly impaired the massive induction of *Sgpp1* in T‐lymphoma cells in response to dexamethasone, but had no effect on basal expression of the gene in these cells. Similarly, although *Sgpp1* was up‐regulated in response to deletion of Runx1 in B‐lymphoma cells, the differential was more marked in the presence of dexamethasone. Replacement with ectopic Runx1 reversed this effect, but not when the DNA or Cbfβ cofactor binding functions were disabled, indicating that suppression most likely operates through the previously mapped binding site in the *Sgpp1* promoter [Kilbey et al., 2010].

Dexamethasone is a synthetic glucocorticoid that induces ceramide and sphingosine to promote apoptosis in mouse thymocytes in a manner that mimics the normal process of T cell selection in the thymus [Cifone et al., 1999; Lepine et al., 2004]. Previous studies in primary mouse thymocytes identified changes in *Runx1*, *Nr3c1*, *Ugcg*, and *Sgpp1* as early consequences of dexamethasone treatment compatible with ceramide‐induced apoptosis [Bianchini et al., 2006]. Our studies in lymphoma cells partially recapitulate these findings, and indicate a causal chain in which down‐regulation of Runx1 is proximal to the other changes and reversed by ectopic expression. Moreover, we have identified positive regulation of Ugcg and repression of Sgpp1 as consistent Runx1 functions. An effector role for Sgpp1 in the depletion of S1P and concomitant dexamethasone‐induced death is further supported by shRNA knock down of *Sgpp1* which, although far from complete, showed a significant sparing effect.

The importance of S1P for survival and drug resistance has been well documented in hematological malignancies but is more commonly attributed to deregulation of Sphk1 or Ugcg rather than to reduced levels of Sgpp1 [Marfe et al., 2011; Casson et al., 2013]. However, the fundamental importance of *Sgpp1* is underlined by the neonatal lethality of gene deletion in mice [Allende et al., 2013] and the effects of modulating expression on cell growth and survival in response to cellular stress. For example, depletion of Sgpp1 promotes ER stress‐induced autophagy in eukaryotic cell lines [Lepine et al., 2011] and thermo tolerance in yeast [Mao et al., 1999], whereas ectopic expression of *Sgpp1* promotes apoptosis and accumulation of intracellular ceramide in NIH3T3 and HEK293 fibroblasts [Le Stunff et al., 2002]. The relevance of *SGPP1* in other cancer types is suggested by its down‐regulation in therapy‐resistant ovarian and prostate cancers [Helleman et al., 2006; Huang et al., 2013].

The observation that ectopic *Runx1* suppresses glucocorticoid‐induced death and that deletion of endogenous *Runx1* has the opposite effect also helps to elucidate the oncogenic consequences of *Runx* over‐expression in lymphoid cells [Stewart et al., 2002, 1997; Wotton et al., 2002]. Moreover, glucocorticoids comprise key components of multi‐drug regimens for the treatment of acute lymphoid malignancies, where resistance is common but the underlying in vivo mechanisms poorly understood [Bhadri et al., 2012]. Our results predict that perturbations in expression of *RUNX* family members in human leukemia will be relevant to cell survival and therapy resistance. In support of this hypothesis, human t(8;21) leukemias that carry only a single functional *RUNX1* allele are unique among AMLs in their sensitivity to glucocorticoids [Miyoshi et al., 1997]. Moreover, relapsing t(12;21) leukemias harboring the *TEL‐RUNX1* fusion display mutually exclusive gains of extra wild‐type RUNX1 on chromosome 21 and losses of the glucocorticoid receptor Nr3c1. In light of these and our observations reported here it will therefore be interesting to test the synergistic potential of glucocorticoids and inhibi tors of *RUNX‐CBFβ* functions [Illendula et al., 2016] or their key downstream targets in sphingolipid metabolism [Edmonds et al., 2011] in the growing number of cancers where the RUNX genes are implicated as oncogenic drivers [Ito et al., 2015].
